# Early Postoperative Low Expression of RAD50 in Rectal Cancer Patients Associates with Disease-Free Survival

**DOI:** 10.3390/cancers9120163

**Published:** 2017-11-30

**Authors:** Vincent Ho, Liping Chung, Amandeep Singh, Vivienne Lea, Maxine Revoltar, Stephanie H. Lim, Thein-Ga Tut, Weng Ng, Mark Lee, Paul de Souza, Joo-Shik Shin, Cheok Soon Lee

**Affiliations:** 1 School of Medicine, Western Sydney University, Penrith, NSW 2751, Australia; liping.chung@westernsydney.edu.au (L.C.); maxine.revoltar@gmail.com (M.R.); tut.theinga@gmail.com (T.-G.T.); P.DeSouza@westernsydney.edu.au (P.d.S.); soon.lee@westernsydney.edu.au (C.S.L.); 2 Ingham Institute for Applied Medical Research, Liverpool, NSW 2170, Australia; stephanie.lim@sswahs.nsw.gov.au; 3 Department of Anatomical Pathology, Liverpool Hospital, Liverpool, NSW 2170, Australia; Amandeep.Singh@sswahs.nsw.gov.au (A.S.); Vivienne.Lea@sswahs.nsw.gov.au (V.L.); 4 Macarthur Cancer Therapy Centre, Campbelltown Hospital, NSW 2560, Australia; 5 Discipline of Medical Oncology, School of Medicine, Western Sydney University, Liverpool, NSW 2170, Australia; 6 Department of Medical Oncology, Liverpool Hospital, Liverpool, NSW 2170, Australia; Weng.Ng@sswahs.nsw.gov.au; 7 Department of Radiation Oncology, Liverpool Hospital, Liverpool, NSW 2170, Australia; Mark.Lee@sswahs.nsw.gov.au; 8 Tissue Pathology and Diagnostic Oncology, Royal Prince Alfred Hospital, Camperdown, NSW 2050, Australia; J.Shin@westernsydney.edu.au; 9 Discipline of Pathology, School of Medicine, Western Sydney University, Campbelltown, NSW 2560, Australia

**Keywords:** RAD50, DNA damage response, rectal cancer, prognosis, biomarkers

## Abstract

Background: Molecular biomarkers have the potential to predict response to the treatment of rectal cancer. In this study, we aimed to evaluate the prognostic and clinicopathological implication of RAD50 (DNA repair protein RAD50 homolog) expression in rectal cancer. Methods: A total of 266 rectal cancer patients who underwent surgery and received chemo- and radiotherapy between 2000 and 2011 were involved in the study. Postoperative RAD50 expression was determined by immunohistochemistry in surgical samples (*n* = 266). Results: Using Kaplan–Meier survival analysis, we found that low RAD50 expression in postoperative samples was associated with worse disease free survival (*p* = 0.001) and overall survival (*p* < 0.001) in early stage/low-grade tumors. In a comparison of patients with low vs. high RAD50 expression, we found that low levels of postoperative RAD50 expression in rectal cancer tissues were significantly associated with perineural invasion (*p* = 0.002). Conclusion: Expression of RAD50 in rectal cancer may serve as a prognostic biomarker for long-term survival of patients with perineural invasion-positive tumors and for potential use in early stage and low-grade rectal cancer assessment.

## 1. Introduction

As the second most common cause of cancer-related death [1], colorectal cancer (CRC) represents a major worldwide health burden. CRC is the third most commonly diagnosed cancer in men and the second in women, with the highest prevalence in Australasia, the Asia-Pacific, Western Europe, and North America [1]. In the United States in 2016, there were approximately 134,490 new cases of CRC (representing 8% of all new cancer cases), and 49,190 estimated deaths [2].

Surgical resection combined with chemotherapy (with or without radiotherapy) is the standard CRC treatment [3,4,5]. Due to local anatomy, surgery alone is usually insufficient to manage patients with rectal cancer; limited operative access, the distance between the tumor border and the mesorectal fascia, and vicinity to the pelvic organs all contribute to recurrence [6]. Additionally, rectal cancer patients consistently have poorer survival outcomes than those with colon cancer [7]. When compared to surgery alone, the use of preoperative (neoadjuvant) radiotherapy significantly reduces the risk of recurrence and improves overall survival (OS) and cancer-specific survival [8,9,10]. 

The DNA damage response (DDR) signaling pathways identify and repair DNA damage [11]. Cells with defective DDR are unable to repair radiation-induced DNA double-strand breaks and therefore have increased sensitivity to radiotherapy. Hence, molecules that are involved in DDR are excellent candidates for radiosensitivity biomarkers [11]. Importantly, abnormalities in DDR mechanisms are linked to carcinogenesis in many human cancers, including CRC. In particular, mismatch repair (MMR) proteins recognize and repair single-nucleotide mismatches in microsatellite sequences that have escaped DNA polymerase proofreading [12]. Approximately 15% of CRCs have mutations in MMR proteins, causing error accumulation during DNA replication and recombination, which result in microsatellite instability (MSI) [12]. The MSI tumor phenotype is of potential clinical significance as a predictor of therapeutic efficacy and prognosis; for example, compared with microsatellite-stable (MSS) tumors with a functional MMR system, MSI tumors have better prognosis [13] but exhibit variable treatment outcomes in response to DNA-targeting chemotherapeutic agents [14].

The MRN complex comprising of DNA repair proteins MRE11, RAD50 and NBS1 (MRE11/RAD50/NBS1) detects and repairs double-strand breaks, and is therefore critical for maintaining genomic integrity and suppressing tumor progression [15,16,17]. Several studies have shown that MRE11 and/or RAD50 mutations often occur in MSI CRC [14,18]. Furthermore, RAD50 is more commonly overexpressed in MSS tumors, while it is more often mutated in MSI tumors [19]. Similarly, MRE11 is a valuable prognostic marker in CRC [20], and its deficiency is associated with improved OS and disease-free survival (DFS) in patients with stage III colon cancer, irrespective of treatment [21]. 

Together with the radiosensitivity index [11,22], MRE11 has been the most studied MRN complex component, and its prognostic value has been assessed in different cancers [15,23], including CRC [18,20,24,25]. However, to date, there is no data available on RAD50 as a specific biomarker in rectal cancer. A recent study evaluated all three MRN proteins together with clinicopathological factors in low-grade epithelial ovarian cancer, and found that RAD50 loss occurred in 10% of cases and was associated with MMR deficiency [26]. Based on the key role played by RAD50 in DNA damage repair [11], we hypothesized that different expression levels of this protein might impact upon treatment outcomes.

In this study, we evaluated the role of RAD50 as a potential prognostic marker in patients with incident cases of rectal cancer treated with surgery and radiotherapy. We correlated postoperative RAD50 tumor expression with patient DFS and OS. Additionally, we assessed the influence of RAD50 expression on other relevant tumor clinico-histopathological variables such as perineural invasion.

## 2. Results

### 2.1. Study Population

A total of 266 patients were included in this study; 176 (66.2%) were male, 90 (33.8%) were female, and the median age was 72 years (range: 35–100 years) (Table 1). Of 266 patients, 77 (31.3%) were treated with radiotherapy, 55 of which (71.4%) received preoperative therapy. Patients were followed for a median period of 3.16 years (range: 0−12.6 years), and the median time to death was 2.5 years after surgery (range: 0−11.1 years).

### 2.2. Association between RAD50 Expression and Clinicopathological Features and Prognosis

**Figure 1:**
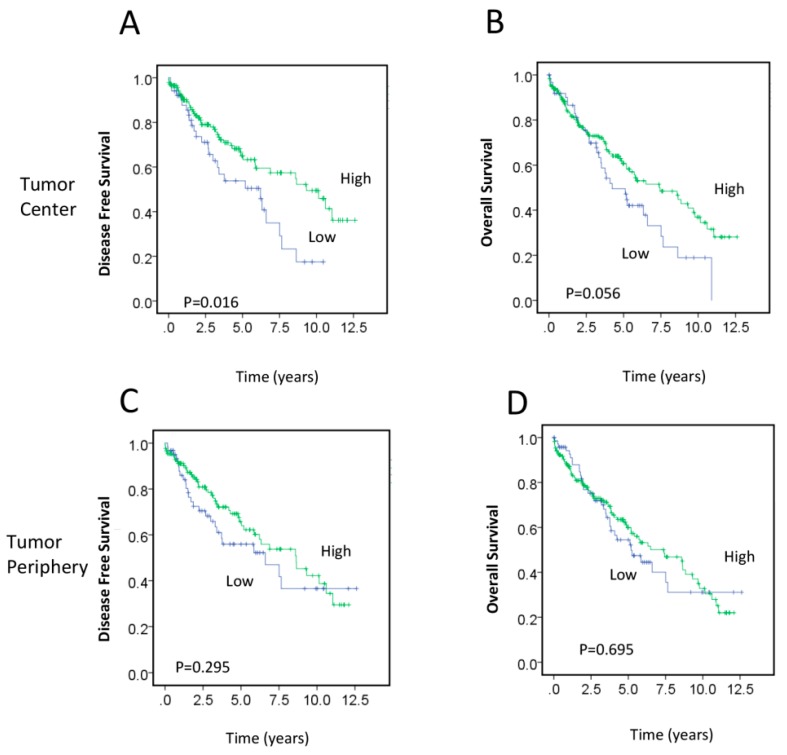
Association between postoperative RAD50 (DNA repair protein RAD50 homolog) expression in the TC (tumor core) and TP (tumor periphery) and survival. (**A**–**D**) Kaplan–Meier survival analysis illustrating DFS (disease free survival) (**A**,**C**) and OS (overall survival) (**B**,**D**) of patients with RAD50 expression in the TC (**A**,**B**) and TP (**C**,**D**). Blue lines represent patients with low RAD50 expression and green lines represent patients with high RAD50 expression.

The association between postoperative RAD50 expression and clinicopathological characteristics is presented in Table 2. Low RAD50 expression levels in the tumor periphery (TP) were significantly associated with adjuvant therapy treatment (*p* = 0.04). There were no significant differences in age, sex, histological tumor stage, lymph node involvement, metastasis, vascular invasion, or perineural invasion between patients with low and high RAD50 expression. Kaplan–Meier survival analysis demonstrated that low RAD50 expression in the tumor center (TC) was significantly associated with worse DFS (*p* = 0.016; Figure 1A), whereas the association of RAD50 expression and OS was of borderline significance (*p* = 0.056; Figure 1B). No significant difference in survival was seen between patients with high or low RAD50 expression in the TP (DFS, *p* = 0.295; OS, *p* = 0.695) (Figure 1C,D). 

To explore the association between RAD50 scores and clinical prognosis, the samples were further categorized into a high expression group (score range: 6–12), a medium-low expression group (score range: 4–<6), and a very low expression group (score range: 0–<4). Kaplan–Meier survival analysis revealed that very low RAD50 expression in the TC was significantly associated with worse DFS (*p* = 0.031) and decreased OS (*p* = 0.044), indicating that the loss of RAD50 expression would possibly increase tumor aggressiveness (Figure S1A,B). No significant change was found between patients with high, medium-low, or/and very low RAD50 expression in the TP (DFS, *p* = 0.111; OS, *p* = 0.638; Figure S1C,D).

We next investigated the status of the MMR pathway in patient samples by evaluating the association of MMR proteins with RAD50 expression. All cases were positive for MLH1 (MutL protein homolog 1) and MSH2 (MutS protein homolog 2) expression; therefore, none of the cases were classified as MSI-high (MMR-negative). Expression of MSH6 (MutS protein homolog 6) and PMS2 (PMS1 homolog 2) were negative in 2/253 (0.8%) and 9/253 (3.6%) cases, respectively. Additionally, no significant associations of RAD50 and MSH6 expression in either TC or TP samples were found. Finally, low postoperative RAD50 expression was significantly associated with PMS2 expression in the TC (*p* = 0.04), but not in the TP (*p* = 0.36) (Table 2).

**Figure 2:**
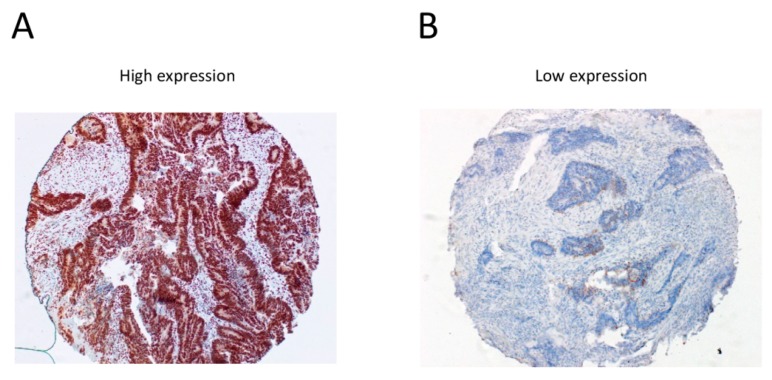

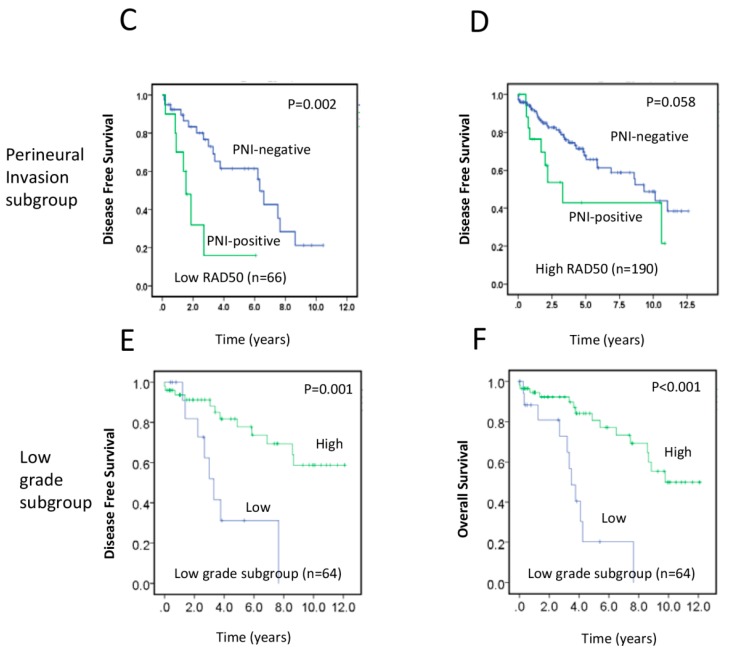
Correlation between RAD50 expression and perineural invasion (PNI) and survival in early stage rectal cancers. (**A**,**B**) Representative immunohistochemical staining of RAD50 in rectal cancer samples (high versus low expression), images were taken at 10× magnification; (**C**,**D**) Kaplan–Meier survival analysis of DFS in low RAD50 expression (**C**) and high RAD50 expression (**D**) groups with (green line) or without (blue line) perineural invasion; (**E**,**F**) Kaplan–Meier survival analysis illustrating the relationship of RAD50 expression with DFS (**E**) and OS (**F**) in low-grade (G1–2) with early tumor stage (T1–2) subgroup. The analyses were divided into four subgroups including the low-grade with early stage tumors (G1–2, T1–2, *n* = 64), high-grade with early stage tumors (G3, T1–2, *n* = 4), low-grade with late stage tumors (G1–2, T3–4, *n* = 113), and high-grade with late stage tumors (G3, T3–4, *n* = 8).

We found that low expression of RAD50 in the TC (HR (hazard ratio) = 0.552, 95% CI 0.339–0.899, *p* = 0.017; univariate Cox regression) was significantly associated with reduced DFS (Table 3). Representative immunohistochemical staining of high and low RAD50 expression in rectal cancer tissues are shown in Figure 2A,B. Additionally, using multivariate Cox analysis, we found that RAD50 expression (HR = 0.567, 95% CI 0.345–0.931, *p* = 0.025) and perineural invasion (HR = 2.364, 95% CI 1.343–4.162, *p* = 0.003) remained significantly associated with DFS (Table 3). However, in multivariate Cox analysis with OS, perineural invasion (HR = 1.701, 95% CI 1.036–2.792, *p* = 0.036) remained significantly associated with OS, but not with tumor RAD50 expression (HR = 0.712, 95% CI 0.462–1.095, *p* = 0.122) (Table S1). Finally, using Kaplan–Meier analysis of DFS to compare RAD50 low and high expression groups with tumors classified by perineural invasion status, we found that low levels of RAD50 expression in rectal cancer tissues were significantly associated with perineural invasion (Figure 2C,D), indicating that low RAD50 in the context of PNI (perineural invasion) is a marker of poor prognosis. 

### 2.3. RAD50 Expression as a Putative Prognostic Factor for Early Stage Rectal Cancer

The DFS of rectal cancer patients with low RAD50 expression was worse than the DFS of patients with high RAD50 expression. When patients were grouped into early tumor stage (T1–2) and low-grade (G1–2) subgroups, low expression of RAD50 was associated with decreased DFS (*p* = 0.001) (Figure 2E), indicating that RAD50 may be a useful prognostic biomarker for early tumor stage and low-grade subgroups. Similarly, low RAD50 expression in early tumor stage and low-grade tumor subgroups was significantly associated with worse OS (*p* < 0.001) (Figure 2F). Additional Cox regression analyses confirmed that expression of RAD50 in early tumor stage and low-grade subgroups significantly correlated with DFS (HR = 0.218, 95% CI 0.084–0.570, *p* = 0.002) (Table 3).

## 3. Discussion

Colon and rectal cancers are often considered as a single entity and are treated with similar approaches. However, rectal cancers, particularly localized rectal cancers, may benefit from a different approach. In this study, we evaluated RAD50 expression by immunohistochemistry in 266 rectal cancer samples, taking into account several clinicopathological characteristics, as well as the type of treatment received. We discovered that low levels of RAD50 expression at early tumor stages and low-grade tumor subgroups were significantly associated with worse DFS and OS, indicating a relationship between postoperative tumor expression of RAD50 and prognosis. 

Long-term clinical trials [27,28] and several meta-analyses [8,10,29] have confirmed that neoadjuvant radiotherapy plus surgery reduces the rate of local recurrence and increases the survival rate of rectal cancer patients, benefits that outweigh the associated side effects. Patients with rectal cancer can have variable responses to radiotherapy; therefore, the identification of rectal cancer-specific radiosensitivity and prognostic markers would enable targeted therapeutic decisions, which would likely improve treatment results and survival rates. For example, it would be useful to identify biomarkers that discriminate between patients who are likely to benefit from neoadjuvant (preoperative) radiotherapy and those who would respond better to adjuvant (postoperative) radiotherapy. 

Based on this premise, we investigated RAD50 as a prognostic biomarker in rectal cancer. Together with MRE11 and NBS1, RAD50 is part of the MRN complex, which is heavily involved in maintenance of genomic integrity and tumor suppression [15]. An intact MRN complex predicts a good response to treatment in patients with early breast cancer [30], whereas mutations in MRE11, NBS1, or RAD50 have been linked to increased risk of cancer, including sporadic and familial cancers [17].

We correlated the clinicopathological characteristics and treatment type with long-term (DFS and OS) radiotherapy responses. Specifically, the results from univariate and multivariate analyses showed that worse DFS outcomes were associated not only with low RAD50 expression in the TC, but also with perineural invasion. These data indicate that postoperative RAD50 expression predicts long-term survival if evaluated with other tumor-related clinicopathological features.

We found that low postoperative RAD50 expression was associated with reduced DFS and OS in early tumor stage (T1–2) and low-grade (G1–2) patient subgroups, supporting the potential use of RAD50 as an early prognostic biomarker in rectal cancer. The role of RAD50 has also been evaluated in CRC. Of interest, Gao et al. [19] demonstrated that RAD50 expression is weak in MSI CRCs and is not associated with clinicopathological characteristics. Conversely, they observed increased RAD50 expression in early stage primary MSS CRCs. Interestingly, they found that the oncogenic RAD50 frameshift mutation (A)^9^ occurred in MSI, but not in MSS CRCs, which suggests that RAD50 might play different roles in these CRC phenotypic subtypes [19].

Importantly, RAD50 was identified as a prognostic biomarker for colorectal mucinous adenocarcinoma (10–15% of all cases) through an integrated analysis of genetic and epigenetic features [31]. The authors of this study showed that weak RAD50 expression was associated with poor prognosis in patients with MSS CRC, and postulated that increased RAD50 expression in MSS CRC could be a tumor suppressive cellular response to prevent further tumor progression [31]. Based on these reports, further investigation of the molecular role of RAD50 in MSI and MSS CRCs could help inform patient response to therapy. 

A limitation of the study was that we focused on individual TNM (tumor, node and metastases) factors rather than overall TNM staging. The current study involved two centers with limited numbers of patients that could have been influenced by our local experience in rectal cancer management. Thus, larger, multicenter prospective studies are needed.

## 4. Materials and Methods

### 4.1. Patients

The study was approved by the South Western Sydney Local Health District Human Research Ethics Committee (HREC Reference: HREC/14/LPOOL/186; project number 14/103). Specimens were collected from 266 patients who underwent surgery and radiotherapy for rectal cancer from 2000–2011. Patients were treated with either a 25 Gy dose administered in five treatment fractions, or a 50.4 Gy dose administered in 28 fractions; both treatment groups received 5-fluorouracil-based chemotherapy. Surgery consisted of total mesorectal excision, as well as anterior or abdominoperineal resection. Follow-up included clinic visits, blood tests, colonoscopy, and imaging based on the recommendation of the treating specialist.

### 4.2. Response and Outcomes of Interest

Short-term response to radiotherapy was measured by TRG (tumor regression grade) according to the 7th edition of the American Joint Committee on Cancer manual [32], which describes a scale of 0–3: 0 represents complete response without any viable malignant cells; 1 is a moderate response with small groups of malignant cells; 2 is a minimal response with fibrosis outgrowing residual malignancy; and 3 is a poor response with residual malignancy. Patients categorized with a TRG of 0 and 1 were considered responders, while patients categorized with a TRG of 2 and 3 were considered non-responders. Variables included age, sex, pathological TNM stage, tumor grade, vascular invasion, perineural invasion, the level of tumor-infiltrating lymphocytes, and treatment. Outcomes were DFS, OS, and histologic TRG in the resected bowel. Long-term outcomes were assessed using Kaplan–Meier curves for DFS and OS. DFS was defined as the time from diagnosis to first recurrence, and OS was defined as the time from diagnosis to the last follow-up point or death. 

### 4.3. Sample Preparation and Tissue Microarrays

Archival formalin-fixed, paraffin-embedded tissue blocks from pre- and postoperative rectal cancer tumors were retrieved for each patient, and two cores (1 mm in diameter) were obtained from each of five different sampling sites: tumor center (TC); tumor periphery at invasive edge (TP); normal mucosa close/adjacent to the tumor; normal mucosa well away from the tumor; and the involved lymph nodes. The corresponding hematoxylin and eosin sections were reviewed to localize the most representative areas of tumor and normal colorectal mucosa in tissue samples. These samples were then transferred into pre-drilled wells in tissue microarray blocks using the Beecher Manual Tissue Microarrayer (Beecher Instruments Inc., Sun Prairie, WI, USA), which were mounted on slides for immunohistochemical analysis. 

### 4.4. Immunohistochemistry

Slide sections taken from the tissue microarrays were deparaffinized in xylene and rehydrated in a graded ethanol series. RAD50 antigen retrieval was performed with the Envision^TM^ FLEX Target Retrieval Solution, pH 9.0, in a 98 °C water bath for 45 min. This was followed by incubation with Envision^TM^ FLEX Peroxidase-Blocking Reagent (Dako, Glostrup, Denmark) for 5 min at room temperature to block endogenous peroxidases. Slides were then incubated with a monoclonal anti-RAD50 primary antibody [13B3/2C6] (1:400 dilution, Abcam #ab89; Cambridge, UK) for 60 min at room temperature. Following antigen retrieval, primary antibodies against MSH6 (1:100), and PMS2 (1:100) (all from Dako, Glostrup, Denmark) were applied for 15 min on board the Dako Autostainer (Dako, Glostrup, Denmark) in 1 mM EDTA (Ethylenediaminetetraacetic acid) buffer, pH 8.0. After washing with Tris-buffered saline with Tween-20, slides were incubated for 15 min with DAKO mouse linker, rinsed, and incubated for 30 min with an anti-mouse secondary antibody. A mixture of Envision^TM^ FLEX DAB + Chromogen DM827 and Envision^TM^ FLEX Substrate Buffer DM823 (Dako, Glostrup, Denmark) was used as a substrate for development. Finally, slides were counterstained with hematoxylin, washed with cold water, and then dipped 10 times in Scott’s Bluing solution (Thermo Fisher Scientific, North Ryde, NSW, Australia). Slides were rinsed immediately with cold water before dehydration and mounting.

Two independent pathologists recorded the intensity and percentage of positive immunohistochemical staining in each sample. RAD50 expression was scored as the product of the staining percentage and intensity as previously described [20]. Intensity was graded as follows: 0, negative; 1, weak; 2, moderate; or 3, strong. The percentage of positive cells was graded as follows: 0 (<5%), 1 (5–25%), 2 (26–50%), 3 (51–75%), or 4 (>75%). These two measures were multiplied to obtain weighted scores ranging from 0 to 12. All tumor samples were categorized into either a low expression group (score range: 0–<6) or a high expression group (score range: 6–12).

### 4.5. Statistical Analysis

Statistical analysis was performed with SPSS for Windows 22.0 (IBM Corporation, Armonk, NY, USA). Survival analysis was conducted for the entire cohort. In addition, further subgroup analysis was conducted for early tumor stage and low-grade tumors as covariates. Univariate and multivariate analyses were performed using Kaplan–Meier curves and Cox’s proportional hazards survival modeling for RAD50 protein expression from the cancer core and periphery. Covariates were sex, age, TNM stage, tumor grade, vascular invasion, perineural invasion, chemotherapy and radiotherapy, TRG, MSH6, and PMS2. Univariate analysis was performed using the Mann–Whitney U test. *p* < 0.05 was considered statistically significant.

## 5. Conclusions

The status of RAD50 expression may have treatment implications for rectal cancer, particularly for predicting long-term survival of patients with perineural invasion and for potential use in early stage and low-grade rectal cancer assessment. Further investigation of RAD50 in larger populations and the identification of new biomarkers are likely to provide new and effective prognostic tools for rectal cancer management.

## Figures and Tables

**Table 1:** Patient characteristics.

Variables	All Patients (%)
Total, *n*	266
Age median	72
Sex	
Male	176/266 (66.2)
Female	90/266 (33.8)
Tumor stage	
T1–2	88/266 (33.1)
T3–4	172/266 (66.9)
Node stage	
N0	140/259 (54.1)
N1–2	119/259 (55.9)
Metastasis stage	
M0	223/240 (92.9)
M1	17/240 (7.1)
Histological Grade	
1–2	246/266 (92.5)
3	20/266 (7.5)
Vascular invasion	
Absent	201/263 (76.4)
Present	62/263 (23.6)
Perineural invasion	
Absent	220/263 (83.7)
Present	43/263 (16.3)
Radiotherapy	
Total	77/246 (31.3)
Neoadjuvant	55/77 (71.4)
Adjuvant	22/77 (28.6)

**Table 2:** Associations between RAD50 protein expression in the tumor center and tumor periphery and clinicohistopathological data.

Variables	Subgroups	Tumor Center			Tumor Periphery		
		Low (%)	High (%)	*p* Value	Low (%)	High (%)	*p* Value
Sex	Male	26.3	73.7	0.98	29.6	70.4	0.42
	Female	26.1	73.9		34.4	65.6	
Age	≤72	28.8	71.2	0.39	30.6	69.4	0.82
	>72	24.1	75.9		31.9	68.1	
Tumor stage	T1–2	23.5	76.5	0.65	32.1	67.9	0.68
	T3–4	26.2	73.8		29.6	70.4	
Node stage	Negative	26.5	73.5	0.67	31.1	68.9	0.95
	Positive	24.1	75.9		30.8	69.2	
Metastasis stage	M0	25.5	74.5	0.72	31.5	68.5	0.09
	M1	29.4	70.6		11.8	88.2	
Histological Grade	1–2	26.1	73.9	0.88	31.3	68.7	0.98
	3	27.8	72.2		31.6	68.4	
Vascular invasion	Absent	26.3	73.7	0.74	31.9	68.1	0.50
	Present	24.2	75.8		27.4	72.6	
Perineural invasion	Absent	24.7	75.3	0.40	32.2	67.8	0.28
	Present	31.1	68.9		23.8	76.2	
Adjuvant therapy	No	25.2	74.8	0.83	24.8	75.2	0.04
	Yes	26.6	73.4		38.8	61.2	
Neoadjuvant therapy	No	23.8	76.2	0.14	27.1	72.9	0.16
	Yes	34.1	65.9		37.3	62.7	
Tumor regression grade	0–1	50	50	0.14	50	50	0.22
	2–3	28.3	71.7		32.8	67.2	
MSH6	Negative	50.0	50.00	0.43	0	100	0.35
	Positive	25.5	74.5		30.8	69.2	
PMS2	Negative	55.6	44.4	0.04	44.4	55.6	0.36
	Positive	25.5	74.5		30.1	69.9	

RAD50: DNA repair protein RAD50 homolog; MSH6: MutS protein homolog 6; PMS2: PMS1 homolog 2.

**Table 3:** Cox regression analyses of postoperative RAD50 with disease-free survival.

Variables	Univariate			Multivariate		
	HR	95% CI	*p*-Value	HR	95%	*p*-Value
RAD50						
Low	0.552	0.339–0.899	0.017	0.567	0.345–0.931	0.025
High						
Age						
≤72	1.279	0.785–2.086	0.323			
>72						
Sex						
Male	1.074	0.657–1.754	0.776			
Female						
Tumor stage						
T1–2	1.643	0.983–2.747	0.058			
T3–4						
Node stage						
Negative	1.198	0.709–1.827	0.593			
Positive						
Histological Grade						
1–2	1.646	0.712–3.804	0.244			
3						
Vascular invasion						
Absent	1.888	0.650–0.2.171	0.575			
Present						
Perineural invasion						
Absent	2.534	0.373–1.134	0.001	2.364	1.343–4.162	0.003
Present						
Adjuvant therapy						
No	0.65	0.373–1.134	0.129			
Yes						
Neoadjuvant therapy						
No	1.147	0.672–0.957	0.616			
Yes						
T1–2, G1–2, † RAD50				0.218	0.084–0.570	0.002
T3–4, G3, † RAD50				0.401	0.065–2.471	0.324

HR, hazard ratio; CI, confidence interval; TC, tumor center, † denotes interaction.

## Supplementary Materials

The following are available online at http://www.mdpi.com/2072-6694/9/12/163/s1. Figure S1: Comparison of survival curves according to score categories between postoperative RAD50 in rectal cancer tissue and survival. Table S1: Cox regression analyses of postoperative RAD50 with overall survival.
