# Supplementary material for: Early Postoperative Low Expression of RAD50 in Rectal Cancer Patients Associates with Disease-Free Survival
Source: Cancers (Basel). 2017 Nov 30;9(12):163. doi: 10.3390/cancers9120163 (PMC5742811; doi:10.3390/cancers9120163)
Supplement: Supplementary file 1 [file cancers-09-00163-s001.docx]

Supplementary Materials: Early Postoperative Low Expression of RAD50 in Rectal Cancer Patients Associates with Disease-Free Survival

Vincent Ho, Liping Chung, Amandeep Singh, Vivienne Lea, Maxine Revoltar,
Stephanie H. Lim, Thein-Ga Tut, Weng Ng, Mark Lee, Paul de Souza, Joo-Shik Shin and Cheok Soon Lee


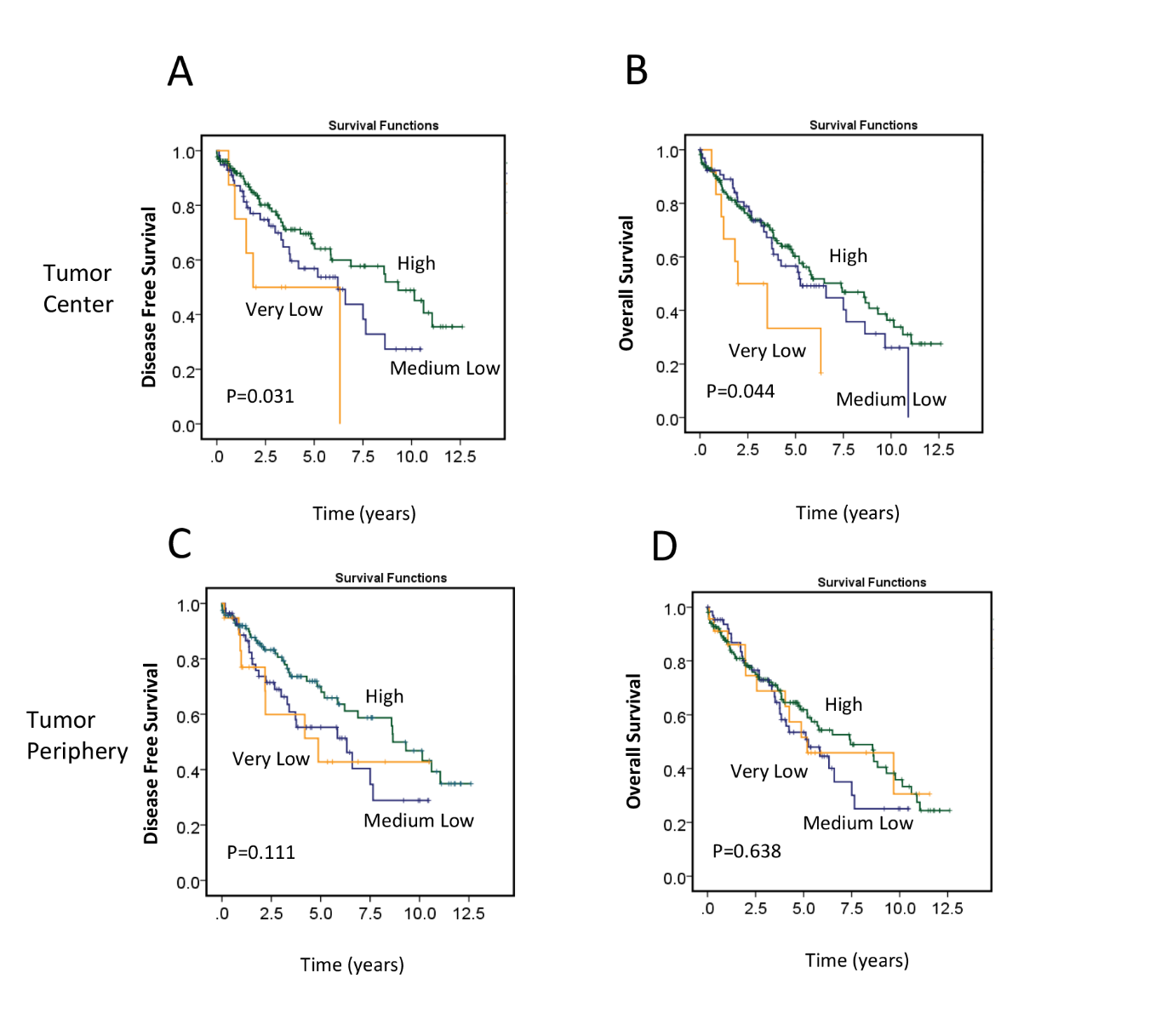


**Figure S1.** Comparison of survival curves according to score categories between postoperative RAD50 in rectal cancer tissues and survival. (**A**–**D**) Kaplan–Meier survival analysis illustrating DFS (disease-free survival) (**A**, **C**) and OS (**B**, **D**) of patients with RAD50 expression in the TC (**A**, **B**) and TP (**C**, **D**). In this analysis, the samples were categorized into a high expression group (score range: 6–12, green line), a medium–low expression group (score range: 4–<6, blue line), and a very low expression group (score range: 0–<4, orange line).

**Table S1.** Cox regression analyses of postoperative RAD50 with overall survival.

| **Variables** | **Univariate** | | | **Multivariate** | | |
| --- | --- | --- | --- | --- | --- | --- |
|  | **HR** | **95% CI** | ***p* Value** | **HR** | **95%** | ***p* Value** |
| RAD50 |  |  |  |  |  |  |
| Low | 0.674 | 0.448–1.012 | 0.047 | 0.712 | 0.462–1.095 | 0.122 |
| High |  |  |  |  |  |  |
| Age |  |  |  |  |  |  |
| ≤72 | 1.334 | 0.897–1.984 | 0.155 |  |  |  |
| >72 |  |  |  |  |  |  |
| Sex |  |  |  |  |  |  |
| Male | 1.092 | 0.734–1.625 | 0.664 |  |  |  |
| Female |  |  |  |  |  |  |
| Tumor stage |  |  |  |  |  |  |
| T1–2 | 1.796 | 1.158–2.786 | 0.001 | 1.382 | 0.858–2.226 | 0.183 |
| T3–4 |  |  |  |  |  |  |
| Node stage |  |  |  |  |  |  |
| Negative | 1.454 | 0.987–2.140 | 0.058 |  |  |  |
| Positive |  |  |  |  |  |  |
| Grade |  |  |  |  |  |  |
| 1–2 | 1.561 | 0.836–2.916 | 0.162 |  |  |  |
| 3 |  |  |  |  |  |  |
| Vascular invasion |  |  |  |  |  |  |
| Absent | 2.03 | 1.340–3.015 | 0.001 | 1.365 | 0.848–2.196 | 0.200 |
| Present |  |  |  |  |  |  |
| Perineural invasion |  |  |  |  |  |  |
| Absent | 2.48 | 1.594–3.859 | 0.000 | 1.701 | 1.036–2.792 | 0.036 |
| Present |  |  |  |  |  |  |
| Adjuvant therapy |  |  |  |  |  |  |
| No | 0.506 | 0.301–0.850 | 0.09 |  |  |  |
| Yes |  |  |  |  |  |  |
| Neoadjuvant therapy |  |  |  |  |  |  |
| No | 0.891 | 0.550–0.427 | 0.63 |  |  |  |
| Yes |  |  |  |  |  |  |

HR, hazard ratio; CI, confidence interval; RAD50: DNA repair protein RAD50 homolog.
